# The Relationship of Food Addiction With Other Eating Pathologies and Impulsivity: A Case-Control Study

**DOI:** 10.3389/fpsyt.2021.747474

**Published:** 2021-11-26

**Authors:** Ekin Sönmez Güngör, Cengiz Çelebi, Yildiz Akvardar

**Affiliations:** ^1^Department of Psychiatry, Erenköy Mental Health and Neurological Diseases Training and Research Hospital, University of Health Sciences, Istanbul, Turkey; ^2^Department of Psychiatry, Büyükçekmece Mimar Sinan State Hospital, Istanbul, Turkey; ^3^Department of Psychiatry, School of Medicine, Marmara University, Istanbul, Turkey

**Keywords:** body mass index, food addiction, obesity, eating disorders, impulsivity

## Abstract

The concept of food addiction (FA) has become central in recent years in understanding the psychological etiology of obesity. In this matched case-control study from Turkey, it was aimed to examine the prevalence of FA and related risk factors in four consecutive body mass index (BMI) categories. The case group consisted of pre-operative bariatric surgery patients with BMI over 35.0 kg/m^2^ (*n* = 40) and the control group was composed of age- and gender- matching individuals from the other categories, namely obese (*n* = 35), overweight (*n* = 40), and normal weight (*n* = 40). The Yale Food Addiction Scale (YFAS) and a standardized clinical interview using the DSM-5 substance use disorders criteria adopted for FA, the Eating Disorder Examination Questionnaire (EDEQ) and the Barratt Impulsivity Scale (BIS-11) were used as assessment instruments. It was found that FA was significantly associated with more serious eating pathologies, more frequent weight-cycling and earlier onset of dieting, higher impulsivity, and higher BMI. Motor and total impulsivity scores showed a positive albeit week correlation with the severity of FA but no significant correlation with BMI, indicating a relationship between impulsivity and weight gain in some but not all individuals. The severity of FA predicted the increase in BMI. Our findings suggest that FA is associated with weight gain in a group of individuals, plausibly through impulsive overeating. Emphasis on FA and its clinical implications such as addiction-based treatments may improve outcomes in obesity and facilitate health promotion.

## Introduction

Obesity is an escalating epidemic in wealthy countries as well as in developing countries and a major cause of preventable deaths worldwide (1, 2). According to a report published by the World Health Organization (WHO) in 2016, more than 1.9 billion adults were overweight (39% of the world population) and of these, over 650 million (13%) were obese (3). As reported in 2019, more than half of the population in 34 out of 36 OECD member countries is overweight and almost one in four people are obese (4). The low rate of success of treatments to prevent or reverse obesity and the significant rate of relapses indicate that obesity might not only be explained as a metabolic disorder but also as a behavioral alteration (5, 6). Recent clinical and neurobiological findings refer to an addictive process contributing to an elevated body mass index (BMI) (7, 8). Based on these, the concept of food addiction (FA), namely the idea that certain highly-processed and “hyperpalatable” foods may have an addictive potential and that certain eating behaviors might be categorized as an addictive behavior was introduced (9–12). Furthermore, an assessment tool was developed based on the addiction criteria listed in the DSM-IV in 2009 (13).

Within a decade after the introduction of the concept, a growing number of clinical and neurobiological studies discussing FA have been published. Experiments with animals and neuroimaging studies in clinical and general populations have shown that individuals with FA demonstrate differences in the dopaminergic activation and functional connectivity of brain reward circuits, including subcortical regions such as amygdala, insula, nucleus accumbens, and ventral striatum (14–16), and some studies also indicate structural variations in reward circuit elements (17), as well as inhibited control of frontal circuits and executive functions such as planning and decision-making (18). A more detailed look reveals that especially processed foods with added sweeteners and fats, and those with high salt (sodium), fat, and sugar content which are calorie-dense have the greatest addictive potential (14, 19, 20), triggering reward mechanisms of the brain and resulting in a “conditioned hypereating” (21). Food addiction has been linked with not only changes in brain circuits but also with alterations in peripheral intestinal, immune, and metabolic mechanisms (20).

However, a well-agreed definition has not yet been established and there is no consensus on the details of this issue. While some authors strongly advocate for the impact of FA on the development of obesity (9, 10) some other experts find the concept unsupported (22), indicate that there is limited evidence (23), or evaluate it as a misnomer (24). Some researchers argue that this concept might lead to the overmedicalization of eating behaviors (25).

Food addiction has been criticized as lacking distinction from other eating disorders as a diagnosis (14, 23). Studies point to a high rate of overlap between FA and binge eating disorder (BED), which are both related to obesity, and in both of which there is an uncontrolled consumption of food and lack of delaying behavioral reactions to food-related stimuli (26, 27). In order to delineate the FA diagnosis from other eating pathologies, it is crucial to investigate factors associated with FA, such as personality factors including reward-sensitiveness and impulsivity which also contribute to the development of other addictions (28–30), in greater depth to establish a more comprehensive and solid model. Considering the changes in the understanding and evaluation of substance-use disorders in the DSM-5, it has become instructive to compare FA to recently updated diagnostic criteria as well (31). Further studies conducted in various settings are required to promote FA to a diagnostic category of universal validity.

In Turkey, the prevalence of obesity is sharply rising, for all genders, in adults and adolescents (32, 33). When compared with the WHO Europe Zone countries, the prevalence of obesity is slightly lower than the European average in men (21.5%), which is around the average in women (24.5%). However, studies assessing FA as a contributing factor to the development of obesity are relatively scarce (29, 34–37).

The aim of this study was to identify the prevalence of FA in a Turkish sample consisting of different BMI classes and to examine the relationship between FA and BMI, focusing on prominent risk factors in addiction including impulsivity. It was hypothesized that FA is related to higher BMI and more frequent dieting. Moreover, it was hypothesized that among other factors, impulsivity is independently associated with FA and the development of obesity.

## Materials and Methods

This study was designed using a matched case-control model. Considering the reported prevalence of FA in the clinical population (29, 38, 39), a sample size of 40 was calculated using a Raosoft sample size calculator, providing a confidence level of 95%, and a margin of error of 5% (40). Therefore, 40 consecutive morbidly obese patients with a BMI >35.0 kg/m^2^ who were evaluated for bariatric surgery at the Marmara University Hospital between 2016 and 2017 who fulfilled the inclusion criteria were recruited as the case group. The control group consisted of age and gender-matched individuals from three other BMI categories, namely obese (*n* = 35), preobese/overweight (*n* = 40), and normal weight (*n* = 40), who were not seeking treatment. Ethical approval for the study was received from Marmara University School of Medicine Local Ethical Committee (Protocol Number 09.2015.244), in accordance with the Declaration of Helsinki. Participation was voluntary in all groups upon informed consent.

The inclusion criteria for the study were defined as being in the ages 18–65, being literate and complying with the instructions, and agreeing to participate in the study. The exclusion criteria were defined as having a severe decompensated medical condition such as acute myocardial infarction, severe respiratory and/or cardiac failure, cerebrovascular disease, cirrhosis, pregnancy (for women), and intellectual/cognitive disability that would not allow reading and understanding the informed consent and cooperating with the clinician for an interview.

A semi-structured interview was conducted to obtain sociodemographic data, clinical characteristics, and eating habits of the participants. The prevalence of eating disorders and subtypes was assessed using structured clinical interviews conducted by researchers based on the DSM-5 criteria. Eating disorder symptomatology was evaluated using the Turkish version of the Eating Disorder Examination Questionnaire (EDEQ) (41, 42). Food addiction diagnosis was assessed using the Turkish version of the Yale Food Addiction Scale (YFAS) (13, 38) and with the semi-structured clinical interview adopted by the researchers from DSM-5 substance use disorder criteria. Finally, impulsivity was assessed using the Turkish version of the Barratt Impulsivity Scale-11 (BIS-11) (43, 44).

Gender, employment, perceived income, and education were included as sociodemographic variables and assessed using descriptive statistics. Body mass index; accompanying medical disorders, current tobacco and alcohol use, dieting attempts, presence of eating disorders, and BIS-11 scores were assessed as independent variables. Chi-square tests were used for categorical variables. Normality distribution of continuous variables was assessed using the Shapiro-Wilk test and means were compared using Student's *t*-tests. Predicting factors of FA were assessed by logistic regression analyses in which FA diagnosis was the dependent variable and sociodemographic and clinical factors, impulsivity scores, and the presence of an eating disorder diagnosis were the independent variables. Statistical significance was set at p < 0.05. To compare multiple groups, a statistical significance level of 0.0083 was accepted following Bonferroni correction (*k* = 4). SPSS 22.0 was used as a software program to compute the data.

## Results

### Descriptive Characteristics of the Case and Control Groups

The case group, consisted of 40 individuals (32 women, 8 men) with a BMI over 35.0 kg/m^2^ had a mean age of 36.05 ± 8.47 years. The control group of consecutive three BMI categories (normal, preobese, and obese) included individuals with a mean age of 32.50 ± 6.34, 36.10 ± 7.41, and 35.77 ± 6.39 years, respectively. Within the case group, 65.0% (*n* = 26) had a job at the time of the study and the majority were married (*n* = 25, 62.5%). The highest rate of employment (77.5%, *n* = 31) was among the normal BMI group, yet the difference between the groups did not differ significantly. The case and control groups did not differ significantly in terms of the assessed sociodemographic variables (Table 1).

**Table 1:** Mean BMI and sociodemographic characteristics of participants.

	**Normal (*n*:40)**	**Preobese/Overweight (*n*:40)**	**Obese (*n* = 35)**	**Morbid obese (*n* = 40)**		***p***
BMI (mean ± SD)	22.13 ± 1.92	27.22 ± 1.27	32.74 ± 2.59	45.17 ± 4.92		<0.001^*^
Age (mean ± SD)	32.50 ± 6.34	36.10 ± 7.41	35.77 ± 6.39	36.05 ± 8.47		0.06^†^
	Min: 20	Min: 24	Min: 23	Min: 21		
	Max:46	Max:52	Max: 47	Max: 54		
	***n*** **(%)**	***n*** **(%)**	***n*** **(%)**	***n*** **(%)**	***χ*** ^**2**^	***p***
Gender						
Women	33 (82.5)	30 (75.0)	28 (80.0)	32 (80.0)	0.35	0.95
Men	7 (17.5)	10 (25.0)	7 (20.0)	8 (20.0)		
Education						
High School or below	21 (52.5)	26 (65.0)	20 (57.1)	25 (62.5)	1.54	0.67
Graduate or above	19 (47.5)	14 (35.0)	15 (42.9)	15 (37.5)		
Employment						
Currently employed	31 (77.5)	27 (67.5)	25 (71.4)	26 (65.0)	3.98	0.26
Currently unemployed	9 (22.2)	13 (32.5)	10 (28.6)	14 (35.0)		
Marital status						
Non-married	19 (47.5)	9 (22.5)	14 (40.0)	15 (37.5)	5.65	0.12
Married	21 (52.5)	31 (77.5)	21 (60.0)	25 (62.5)		
Perceived economic status						
Low	6 (15.0)	3 (7.5)	2 (5.7)	6 (15.0)	8.96	0.44
Low–Average	18 (45.0)	23 (57.5)	20 (57.1)	16 (40.0)		
High–Average	14 (35.0)	13 (32.5)	13 (37.1)	18 (45.0)		
High	2 (5.0)	1 (2.5)	0 (0.0)	0 (0.0)		

* *Assessed with ANOVA, F_(3, 119)_ = 372.17, p < 0.05*.

† *Assessed with ANOVA, F_(3, 118)_ = 2.51, p = 0.062*.

The distribution of BMIs of participants in four BMI categories were normal. The mean BMIs of each group were as follows: 22.13 ± 1.92 kg/m^2^ (18.80–25.00), 27.22 ± 1.27 kg/m^2^ (25.20–29.80), 32.74 ± 2.59 kg/m^2^ (30.0–38.05), 45.17 ± 4.92 kg/m^2^ (37.80–58.90). Tobacco and alcohol use and family history were similar among the groups (Table 2), however, those with a BMI >30.0 kg/m^2^ (*n* = 75) had a significantly higher rate (63.5%) of obesity in family history, when compared to the rest (χ^2^ = 23.19, *p* < 0.01). The morbidly obese group had a significantly higher rate of accompanying chronic medical disorders, namely diabetes, hypertension, and hyperlipidaemia (25, 20, and 10%, respectively).

**Table 2:** Clinical characteristics of participants.

	**Normal (*n*:40)**	**Preobese/Overweight (*n*:40)**	**Obese (*n* = 35)**	**Morbid obese (*n* = 40)**		
	***n* (%)**	***n* (%)**	***n* (%)**	***n* (%)**	**χ^**2**^**	***p***
Tobacco use						
Yes	10 (25.0)	16 (40.0)	17 (48.6)	20 (50.0)	6.43	0.09
No	30 (75.0)	24 (60.0)	18 (51.4)	20 (50.0)		
Alcohol use						
Yes	0 (0.0)	0 (0.0)	0 (0.0)	3 (7.5)	8.80	0.03
No	40 (100.0)	40 (100.0)	35 (100.0)	37 (92.5)		
Medical disorders						
Yes	6 (15.0)	12 (30.0)	13 (37.1)	28 (70.0)	27.45	<0.001
No	34 (85.0)	28 (70.0)	22 (62.9)	12 (30.0)		
Family history of chronic medical disorders						
Yes	27 (67.5)	30 (75.0)	30 (85.7)	36 (90.0)	7.53	0.06
No	13 (32.5)	10 (25.0)	5 (14.3)	4 (10.0)		
Family history of alcohol use disorders (AUD)						
Yes	9 (22.5)	8 (20.0)	6 (17.1)	5 (12.5)	1.49	0.69
No	31 (77.5)	32 (80.0)	29 (82.9)	35 (87.5)		
Family history of substance use disorders (SUD)						
Yes	0 (0.0)	1 (2.5)	2 (5.7)	2 (5.0)	2.50	0.48
No	40 (100.0)	39 (97.5)	33 (94.3)	38 (95.0)		
Family history of psychiatric disorders (other than AUD and SUD)						
Yes	11 (27.5)	10 (25.0)	8 (22.9)	7 (17.5)	1.22	0.75
No	29 (72.5)	30 (75.0)	27 (77.1)	33 (82.5)		

### Food Addiction Symptomatology

The different BMI groups differed significantly in terms of FA diagnosis by both instruments (Table 3). Food addiction was found to be more prevalent in the two groups with BMI >30 kg/m^2^ (morbid obese, *n* = 40 and obese, *n* = 35) than in the normal (*n* = 40) and overweight (*n* = 40) individuals (*p* < 0.01), as measured by the YFAS (23.8 vs. 0.0%) and DSM-5 clinical interview (57.5 vs. 12.5%). In terms of severe FA as assessed by the DSM-5 (having six or more symptoms), the obesity and morbid obesity group demonstrates 8.88 times higher prevalence than the normal and overweight groups (33.3 vs. 3.7%).

**Table 3:** Food addiction and eating disorders diagnoses and symptomatology and impulsivity in different BMI groups, as assessed by YFAS and DSM-5 clinical interviews, EDEQ, and BIS-11.

	**Normal (*n*:40)**	**Preobese/Overweight (*n*:40)**	**Obese (*n* = 35)**	**Morbid obese (*n* = 40)**		
	***n* (%)**	***n* (%)**	***n* (%)**	***n* (%)**	**χ^**2**^**	***p***
YFAS assessment						
FA (–)	40 (100.0)	40 (100.0)	31 (91.2)	34 (85.0)	11.61	<0.001
FA (+)	0 (0.0)	0 (0.0)	3 (8.8)	6 (15.0)		
DSM-5 FA assessment						
FA (–)	35 (87.5)	35 (87.5)	19 (54.3)	10 (25.0)	48.82	<0.001
FA (+)	5 (12.5)	5 (12.5)	16 (45.7)	30 (75.0)		
Mild	1 (2.5)	2 (5.0)	4 (11.4)	6 (15.0)		
Moderate	2 (5.0)	2 (5.0)	3 (8.6)	8 (20.0)		
Severe	2 (5.0)	1 (2.5)	9 (25.7)	16 (40.0)		
DSM-5 eating disorders						
Not present	33 (82.5)	34 (85.0)	24 (68.5)	14 (35.0)	–	–
AN	0 (0.0)	0 (0.0)	0 (0.0)	0 (0.0)	–	–
BN	1 (2.5)	0 (0.0)	0 (0.0)	1 (2.5)	1.90	0.59
BED	1 (2.5)	2 (5.0)	5 (14.0)	10 (25.0)	12.17	<0.001
Atypical AN	0 (0.0)	0 (0.0)	0 (0.0)	0 (0.0)	–	–
Atypical BN	0 (0.0)	0 (0.0)	1 (2.9)	1 (2.5)	2.18	0.54
Atypical BED	0 (0.0)	3 (7.5)	4 (11.4)	3 (7.5)	4.34	0.23
NES	5 (12.5)	1 (2.5)	1 (2.9)	11 (27.5)	15.72	<0.001
					***χ*** ^**2**^	***p***
					**KW**	
EDEQ scores						
Total	0.65 ± 0.96	1.44 ± 1.01	2.03 ± 1.33	2.40 ± 1.10	48.20	<0.001
Restriction	0.63 ± 1.11	1.34 ± 1.17	1.76 ± 1.46	1.76 ± 1.16	29.30	<0.001
Food concerns	0.36 ± 0.75	0.61 ± 0.64	1.02 ± 1.01	1.31 ± 1.15	28.53	<0.001
Body concerns	0.91 ± 1.12	2.14 ± 1.49	2.93 ± 1.88	3.49 ± 1.70	46.40	<0.001
Weight concerns	0.74 ± 1.03	1.65 ± 1.24	2.41 ± 1.62	3.03 ± 1.38	57.71	<0.001
BIS-11 scores						
Total	59.12 ± 7.44	58.90 ± 8.86	58.32 ±10.20	61.28 ± 8.62	4.74	0.19
Non-planning	26.00 ± 4.63	25.85 ± 4.62	24.97 ± 4.61	26.87 ± 4.75	4.32	0.23
Motor impulsivity	19.22 ± 3.68	19.65 ± 3.68	19.35 ± 4.42	20.76 ± 4.11	3.60	0.31
Attentional imp.	13.90 ± 2.91	13.40 ± 3.24	13.50 ± 3.06	13.64 ± 2.88	1.19	0.76

Food addiction diagnosis by both instruments was associated with a higher rate of chronic medical disorders (χ^2^ = 7.0, *p* < 0.01) and tobacco and alcohol use (χ^2^ = 4.20, *p* = 0.04; χ^2^ = 5.41, *p* = 0.02). Dieting and lifetime number of diet attempts were significantly higher in those with FA (11.22 ± 8.23; median 10) than in those without FA (6.89 ± 7.09; median 4) (*z* = −2.03, *p* = 0.04).

The most prevalent symptoms as assessed by the DSM-5 adopted clinical interview were (i) consumption of food in larger amounts or over a longer period than intended (71.3%), (ii) persistent desire or unsuccessful efforts to cut down or control (70.5%), and (iii) craving (45.1%); all indicating loss of control over food. Likewise, persistent desire or unsuccessful efforts to cut down or control (93.9%), tolerance (49.0%), and consumption despite persistent physical or psychological problems caused or exacerbated by it (46.9%) were the most frequently met criteria in YFAS assessments.

Food addiction severity, as defined by symptom count in both assessments, showed a significant correlation between YFAS (out of 7 criteria) and DSM-5 (out of 11 criteria). Greater FA severity correlated with increased BMI. Linear regression analysis showed that the severity of FA, measured as the DSM-5 symptom count predicted an increase in BMI [*F*_(1.153)_ = 49.095, *p* < 0.01, *R*^2^ = 0.243]. The BIS-11 total and sub-scale scores did not significantly differ among BMI categories (*z* = −1.19, *p* = 0.24; *z* = −1.27, *p* = 0.21; *z* = −0.76, *p* = 0.45; *z* = −0.79, *p* = 0.43, respectively). Motor and total impulsivity scores showed a positive albeit weak correlation with the severity of FA (assessed by symptom count) but no significant correlation with BMI (Table 4).

**Table 4:** Correlation of BMI, FA symptom count, and BIS-11 scores.

	**DSM-5 symptom count**	**YFAS symptom count**	**BIS-11 non-planning**	**BIS-11 motor impulsivity**	**BIS-11 attentional impulsivity**	**BIS-11 Total**
BMI	0.507^*^	0.561^*^	0.084	0.122	−0.019	0.085
DSM-5 symptom count	1	0.631^*^	0.006	0.191^†^	0.89	0.130
YFAS symptom count	0.631^*^	1	0.121	0.258^*^	0.125	0.223^*^
BIS-11 non-planning	0.006	0.121	1	0.341^*^	0.436^*^	0.822^*^
BIS-11 motor impulsivity	0.191^*^	0.258^*^	0.341^*^	1	0.322^*^	0.702^*^
BIS-11 attentional impulsivity	0.089	0.125	0.436^*^	0.322^*^	1	0.702^*^
BIS-11 total	0.130	0.223^*^	0.822^*^	0.702^*^	0.702^*^	1

* *Spearman correlation, p < 0.01*.

† *p < 0.05*.

### Eating Disorder Symptomatology

The morbid obesity group had significantly higher rates of current BED (25.0%) and night eating syndrome (27.5%) diagnoses, as assessed by the DSM-5 criteria (*p* < 0.001). The total and sub-scale scores of the EDEQ were not normally distributed. A Kruskal-Wallis assessment revealed that the total and sub-scale scores of different BMI categories differed significantly, showing that higher BMI was associated with higher EDEQ scores. When the FA and non-FA groups were compared, FA was significantly associated with more severe eating symptomatology as assessed by EDEQ (Table 5).

**Table 5:** Comparison of participants with and without food addiction as assessed by two instruments.

	**DSM-5 Clinical Interview**				**YFAS**			
	**FA (+)**	**FA (–)**	*z* ^*^	***p***	**FA (+)**	**FA (–)**	***z* ^†^**	***p***
	**(*n* = 56)**	**(*n* = 99)**			**(*n* = 9)**	**(*n* = 145)**		
DSM-5 symptom count	5.27 ± 1.93	2.25 ± 1.89	−7.55	<0.01	5.33 ± 1.87	3.17 ± 2.34	−2.61	<0.01
YFAS symptom count	2.83 ± 1.34	1.42 ± 1.06	−6.47	<0.01	4.78 ± 1.30	1.75 ± 1.14	−4.88	<0.01
BIS-11 total	60.90 ± 9.55	58.56 ± 8.25	−1.73	0.83	62.56 ± 9.99	59.20 ± 8.70	−1.10	0.27
BIS-11 non-planning	26.14 ± 4.77	25.83 ± 4.64	−0.67	0.50	25.44 ± 4.33	25.98 ± 4.71	−0.09	0.93
BIS-11 motor impulsivity	20.98 ± 4.75	19.27 ± 3.36	−2.02	0.04	21.56 ± 3.54	25.98 ± 4.71	−1.53	0.13
BIS-11 attentional impulsivity	13.78 ± 2.99	13.45 ± 2.95	−0.69	0.49	15.56 ± 3.17	13.44 ± 2.91	−1.93	0.05
EDEQ total	2.43 ± 1.17	1.16 ± 1.07	−6.10	<0.01	3.07 ± 0.83	1.81 ± 1.50	−3.34	<0.01
EDEQ restriction	1.90 ± 1.33	1.06 ± 1.19	−4.36	<0.01	2.22 ± 1.22	1.30 ± 1.29	−2.28	<0.01
EDEQ eating concerns	1.33 ± 1.02	0.52 ± 0.78	−5.71	<0.01	1.91 ± 0.74	0.74 ± 0.92	−3.64	<0.01
EDEQ body shape concerns	3.52 ± 1.61	1.69 ± 1.60	−6.13	<0.01	4.26 ± 0.87	1.81 ± 1.50	−3.18	<0.01
EDEQ weight concerns	2.98 ± 1.43	1.35 ± 1.31	−6.30	<0.01	3.87 ± 0.83	1.52 ± 1.23	−3.51	<0.01

* *Mann-Whitney U-tests were used as groups did not have a normal distribution*.

† *Mann-Whitney U-tests were used as groups did not have a normal distribution*.

### Comparison of Food Addiction and Eating Disorders

When morbid obesity, FA, and BED diagnoses were examined together, although comorbidities were present, the majority of FA diagnoses (75%) did not meet the diagnostic criteria for BED. In the morbid obesity group, 22.5% had both FA and BED diagnoses. The comorbid group differed from the FA-only group with greater tolerance (χ^2^ = 6.10, *p* = 0.01), failure to fulfill major role obligations (χ^2^ = 9.93, *p* < 0.01), and higher attentional impulsivity scores (*z* = −2.08, *p* = 0.04). On the other hand, the FA-only group differentiated from the comorbid FA and BED group, as they met the two following BED criteria significantly less frequently: (i) repetitive binge eating episodes and a sense of lack of control over eating during the episode, and (ii) feeling disgusted with oneself, depressed, or very guilty after overeating (*p* = 0.02, *p* = 0.06, respectively).

Finally, a logistic regression was performed to ascertain the effects of age, gender, sociodemographic characteristics, eating disorders and impulsivity on the likelihood that participants had FA. The logistic regression model was statistically significant, [χ${}_{\left(11\right)}^{2}$ = 33.46, *p* < 0.05]. The model explained 45.7% (Nagelkerke *R*^2^) of the variance in FA. Women were 6.7 times more likely to exhibit FA than men. The presence of BED (OR: 8.33 %95CI [1.96–22.42]; *p* < 0.05) and higher BIS-11 scores (OR = 1.09 %95CI [1.02–1.23]; *p* = 0.03) independently predicted the diagnosis of FA.

## Discussion

In this case-control study assessing the extent of and related factors with FA in different BMI categories, it was found that FA is associated with a higher BMI, an earlier onset of dieting and more frequent diet-weight gain cycles; as well as higher attentional and motor impulsivity. Moreover, the FA symptom count was positively correlated with BMI. Our findings suggest that FA might play an important role in obesity, through loss of control over food consumption in an addictive manner. Therefore, treating FA might be a useful approach in terms of weight loss.

### The Prevalence of FA Using DSM-IV and DSM-5 Approaches

The prevalence of FA in the morbid obesity group as assessed by YFAS (15.0%) is comparable with that in two studies in the field, in which 15 and 16.9% of bariatric surgery candidates were diagnosed with FA (45, 46). However, there are other studies conducted among individuals undergoing weight-loss surgery in which higher rates such as 21.1% (47), 25.8% (48), 41.7% (49), 53.7% (50), 57.8 (38) were found. This large variation might be due to the self-report nature of YFAS, which is less-objective than a standardized clinical evaluation and that our sample had a lower mean BMI than the aforementioned studies. It is stated that DSM-IV substance dependence diagnosis corresponds to severe substance use disorder of the DSM-5 (51). Given this, the prevalence of FA in the morbid obesity group appears to be 40.0%, implying that DSM-5 criteria might be more permissive in terms of determining FA, whereas YFAS might overlook some cases. Moreover, as YFAS, which is based on the DSM-IV substance dependence criteria does not include craving, which might contribute to lower than actual rates.

Our findings based on the DSM-5 adopted clinical interviews, especially the prevalence of FA in normal-weight and obese individuals, are similar to the findings of the studies conducted using the revised version of YFAS, YFAS 2.0 (52, 53). In accordance with the studies of Meule (54) and Hauck (53), it was found that there was a moderate level correlation between the FA symptom count and BMI (ρ = 0.56) and the FA prevalence was nine times higher in individuals with a BMI >30 kg/m^2^ than in normal-overweight individuals.

### Clinical Characteristics Related With FA

It was found that in the FA group, the dieting onset was earlier and diet-weight gain cycles were more frequent. This also supports that there might be an addictive, relapsing process behind in this subgroup of obese patients, as many other addictions also start during adolescence and early adulthood (55, 56). It is also true that eating pathologies tend to arise during this developmentally sensitive period (57).

Our findings reflect that the impulsivity scores of the case and control groups did not differ significantly from each other. However, significantly higher attentional and motor impulsivity scores were found in the FA group than in the non-FA group, supporting our hypothesis. This might be related to impaired inhibitory control or the so-called negative urgency in these individuals toward specific food products. The findings can be interpreted as impulsivity not playing a direct role in weight gain but rather contributing to obesity in a subgroup of patients who present with FA. A similar mechanism has been implicated in other studies that explore impulsivity and FA. Higher attentional impulsivity (54, 58, 59), negative urgency (60), inability to delay rewards (61), and controlling impulses in negative mood states (62) were found to be related to FA. One study found that impulsivity persists after bariatric surgery (63), which may also be suggestive of impulsivity and FA as the reason behind unsuccessful weight loss outcomes. Neurocognitive studies also suggest deficits in inhibitory functions and dysfunctional frontostriatal circuits leading to eating pathologies and loss of control over food (14).

Tolerance, withdrawal, and craving constitute the most prominent symptoms of FA, whereas in eating disorders, including BED, weight, and body image related concerns are more critical. It was confirmed in several other studies that some FA patients did not meet the BED criteria and vice versa (64, 65). Although loss of control might be the intersecting symptom of the two diagnoses, it was found that BED is related to the binge eating episodes, that is, eating a substantially large amount of food in a very short time. However, in FA, one of the most important aspects of diagnosing substance use disorder the loss of control is observed throughout the day such as grazing, resulting in eating more than intended or planned and higher caloric intake, in a fashion like tobacco consumption. This finding is parallel to the interpretation of Gearhardt and Meule, who evaluated the concept in light of the DSM-5 (66).

As in our study, female gender was repeatedly found to be associated with FA in other studies (10, 60, 67, 68). It is known that women are more likely to self-medicate than men in the acquisition phase of addiction and show a more rapid escalation of use than men, whereas in general substance use disorders are much more common in men (69). The wide availability of hyperpalatable, high-calorie, and inexpensive food and the contextual and social factors that do not necessarily prevent women from consuming these drugs, contrary to that of illicit drugs, might explain why FA is more frequently observed in women than in men.

In our study, risky alcohol use in the morbid obesity group (7.5%) was higher than the general population rates of risky alcohol use reported in other studies from Turkey (70, 71). Higher rates of risky alcohol use might be interpreted as contributing to the development of obesity. This might also mean a predisposition to addiction in this group. Indeed, the higher rate of risky alcohol use in the FA group than in the non-FA group might be related to the cross-addiction phenomenon, which signifies addiction to two or more substances or behaviors, and is frequently observed in substance-related and addictive disorders (72–74).

### Limitations and Strengths of the Study

Our study has some limitations. First, as the study used a cross-sectional method, our findings reflect associations between variables. More prospective and long-term studies, including those which investigate both clinical and neurobiological/hormonal/metabolic correlates of FA are required to better understand the causality. The case and the control groups were not compared for their diet characteristics and food environment which may also play a role in the development of FA. Also, for the classification of body weight, only BMI was used and bioimpedance analysis was not conducted, which would have enabled a more precise comparison, a method that needs to be considered for future studies. The second version of the YFAS, which is prepared in line with the newly established diagnostic criteria for substance use disorder in the DSM-5, was not used, as it had not been validated in Turkish at the time of the study (52). Nevertheless, the contemporary approach to addictive behaviors based on DSM-5 criteria was reflected by means of a clinical interview, which also enabled us to make a complementary evaluation in addition to the self-report assessment by the YFAS. In addition, the inclusion of consecutive BMI groups and comparison of FA-related features provide a more comprehensive understanding of this construct and contribute to the discussions in the field on the validity of FA, which we believe is a strength of this study.

To conclude, our findings support the view that a group of obesity patients demonstrate characteristics of addictive behaviors and FA contributes to the development of obesity, plausibly through impulsive but not-episodic over-eating. However, FA explains only part of the variance in BMI, which is in accordance with the multiple etiologies of obesity. Further neurobiological and clinical studies in different patient groups from different cultural profiles are necessary to address the universal validity of FA and specific treatments targeting this construct, such as brief interventions or other addiction-based treatments such as relapse prevention, which may help improve the treatment outcome in obesity and may facilitate health promotion.

## Data Availability Statement

The raw data supporting the conclusions of this article will be made available by the authors, without undue reservation.

## Ethics Statement

The studies involving human participants were reviewed and approved by Marmara University School of Medicine Local Ethical Committee. The patients/participants provided their written informed consent to participate in this study.

## Author Contributions

ES and YA designed the study and analyzed the data. ES and CÇ collected the data. ES wrote the initial draft. YA supervised the study. All authors commented on the revised version of the manuscript.

## Conflict of Interest

The authors declare that the research was conducted in the absence of any commercial or financial relationships that could be construed as a potential conflict of interest.

## Publisher's Note

All claims expressed in this article are solely those of the authors and do not necessarily represent those of their affiliated organizations, or those of the publisher, the editors and the reviewers. Any product that may be evaluated in this article, or claim that may be made by its manufacturer, is not guaranteed or endorsed by the publisher.
